# An EEG & eye-tracking dataset of ALS patients & healthy people during eye-tracking-based spelling system usage

**DOI:** 10.1038/s41597-024-03501-y

**Published:** 2024-06-22

**Authors:** Thi Duyen Ngo, Hai Dang Kieu, Minh Hoa Nguyen, The Hoang-Anh Nguyen, Van Mao Can, Ba Hung Nguyen, Thanh Ha Le

**Affiliations:** 1 University of Engineering and Technology, Vietnam National University, Hanoi, Vietnam; 2 Vietnam-Korea Institute of Science and Technology, Ministry of Science and Technology, Hanoi, Vietnam; 3 Vietnam Military Medical University, Hanoi, Vietnam

**Keywords:** Neurology, Health care

## Abstract

This research presents a dataset consisting of electroencephalogram and eye tracking recordings obtained from six patients with amyotrophic lateral sclerosis (ALS) in a locked-in state and one hundred seventy healthy individuals. The ALS patients exhibited varying degrees of disease progression, ranging from partial mobility and weakened speech to complete paralysis and loss of speech. Despite these physical impairments, the ALS patients retained good eye function, which allowed them to use a virtual keyboard for communication. Data from ALS patients was recorded multiple times at their homes, while data from healthy individuals was recorded once in a laboratory setting. For each data recording, the experimental design involved nine recording sessions per participant, each corresponding to a common human action or demand. This dataset can serve as a valuable benchmark for several applications, such as improving spelling systems with brain-computer interfaces, investigating motor imagination, exploring motor cortex function, monitoring motor impairment progress in patients undergoing rehabilitation, and studying the effects of ALS on cognitive and motor processes.

## Background & Summary

Amyotrophic Lateral Sclerosis (ALS) is a progressive neurodegenerative disorder that results in paralysis and severely impairs the communication abilities of patients in the advanced stages^1,2^. When patients have intact consciousness and limited motor function, including voluntary eye movement control, muscle twitching or blinking, they are said to be in a locked-in state (LIS)^3,4^. For LIS patients, eye-tracking based augmentative and alternative communication (AAC) technologies are currently the most effective and almost the only means of communication^5,6^. Previous studies have suggested that eye-controlled on-screen keyboards are the most suitable and optimal method to facilitate communication for LIS patients, as they still retain full cognitive capacity, and eye control ability remains relatively intact despite advanced general paralysis^5^.

The most widely used eye-tracking method in AAC systems for individuals in LIS involves the detection and tracking of eye movements^7^. This technology tracks the direction of users’ gaze on a screen or interface, enabling them to select letters, words, or symbols to form messages. Generally, users utilize their eye gaze to select a key on an on-screen keyboard. Gaze-tracking technique enables the user to select any on-screen key by using their eye gaze to determine the position of the selected key and to signal the system to recognize it^8–10^. Building upon this method, an eye-tracking based spelling communication system was developed to support Vietnamese individuals with impaired speech motor function and specifically those with ALS, to communicate by typing text messages via an on-screen keyboard using their eye gaze^11^. The system’s on-screen keyboard has a key layout and a quick typing suggestion mechanism tailored specifically for the Vietnamese language. Six ALS patients with varying degrees of functional impairment, ranging from partial to full paralysis, were able to use the eye-tracking based spelling communication system to select letters to form words and sentences, and thus communicate freely. The study’s design and paradigm are described in detail in the Methods section.

This data descriptor presents electroencephalogram (EEG) and eye-tracking (ET) recordings captured from a total of 176 participants, consisting of six distinct ALS patients and 170 healthy individuals. To provide context, the prevalence of ALS in the United States was reported to be 5,2 per 100.000 persons in 2015, according to the US National ALS Registry^12^. The recordings in the proposed dataset, named the EEGET-ALS dataset^13^, were obtained while the participants utilized the eye-tracking-based spelling communication system, as well as when they performed other requested tasks. More specifically, the EEG recordings were acquired during the execution of specific tasks, such as imagining particular movements or demands and performing those movements, by both ALS patients and healthy participants. For ALS patients, the movements were performed to the extent possible given their functional impairment. The EEG and ET recordings were simultaneously obtained during the use of the eye-tracking based spelling communication system, as participants typed sentences corresponding to the movements or demands.

There have been previous studies with similar objectives, and several EEG and/or ET datasets have been publicly released^14–17^. However, most of these datasets are focused solely on motor imagery or have been recorded with a limited number of subjects. Furthermore, there are only a few published EEG and/or ET datasets available concerning ALS patients, and no datasets containing ET and/or EEG recordings of both ALS patients and healthy individuals have been found. To the best of our knowledge, there are no datasets with the same characteristics as the one reported here in the available open-access specialized repositories^18–20^. It is noteworthy that the proposed EEGET-ALS dataset^13^ contains simultaneous recordings of ET and EEG signals from six distinct ALS patients and 170 different healthy individuals, which were recorded using dedicated devices for each type of signal. These ET and EEG data were captured under identical experimental design from healthy participants and ALS patients in different advanced stages, who were either gradually descending into the LIS state or had been already completely in it. The dataset^13^ highlights a phase in ALS where communication becomes progressively more challenging and eventually impossible without AAC. It includes recordings from ALS patients over a span of months as well as recordings from a large number of healthy individuals. Furthermore, the dataset^13^ contains EEG/ET data relevant to other task imagination and other activities, not just motor imagery. As a result, we believe that the EEGET-ALS dataset^13^ can serve as a valuable benchmark for improving spelling systems with brain-computer interfaces, studying motor imagery, investigating ALS, studying motor cortex function, and examining the progress of patients with motor impairments during rehabilitation, among other applications.

## Methods

The experiment described in this study was approved by the Institutional Review Board in Human Research Dinh Tien Hoang Institute of Medicine, which has the operating codes as IRB-VN02010 issued by Vietnam Ministry of Health & as IRB00010830 and IORG0009080 issued by U.S. Department of Health and Human Services. The study was conducted in accordance with the guidelines established by Dinh Tien Hoang Institute of Medicine. Written informed consent was obtained from all participants, including both healthy individuals and patients or their legal representatives, to permit publication of their data. The methods described here are complementary to an in-depth description of the results derived from this dataset that have been presented in <*Potential Applications>*.

### Participants

To recruit participants for the study, a recruitment notice for voluntary participation was widely disseminated. Subsequently, for those who volunteered, physicians within our research team conducted assessments and selected individuals to participate in data collection. These individuals needed to meet certain criteria: if they were not ALS patients, they had to have normal mobility and no prior history of neurological, psychological, or language disorders; if they were ALS patients, their eyes still had to function well enough to use the eye-tracking-based spelling system.

In this study, the data recording process involved the participation of six distinct ALS patients and 170 healthy individuals. The healthy participants, who had normal mobility and no prior history of neurological, psychological, or language disorders, were between 19 to 70 years of age. Of the ALS patients, four patients had a functional rating scale revised (ALSFRS-R)^21^ score of 0 in the LIS, while the remaining two had a ALSFRS-R score of 1 in the LIS. The participants were identified only by their aliases “id001” through “id170” and “ALS01” through “ALS06”. The data recording process for healthy individuals was conducted once in the laboratory, whereas for each ALS patient, it was performed multiple times at their home. The team visited each ALS patient every few weeks and conducted the data recording depending on their health status and convenience, with each recording time lasting approximately one hour.

### Spelling communication system

**Fig. 1:**
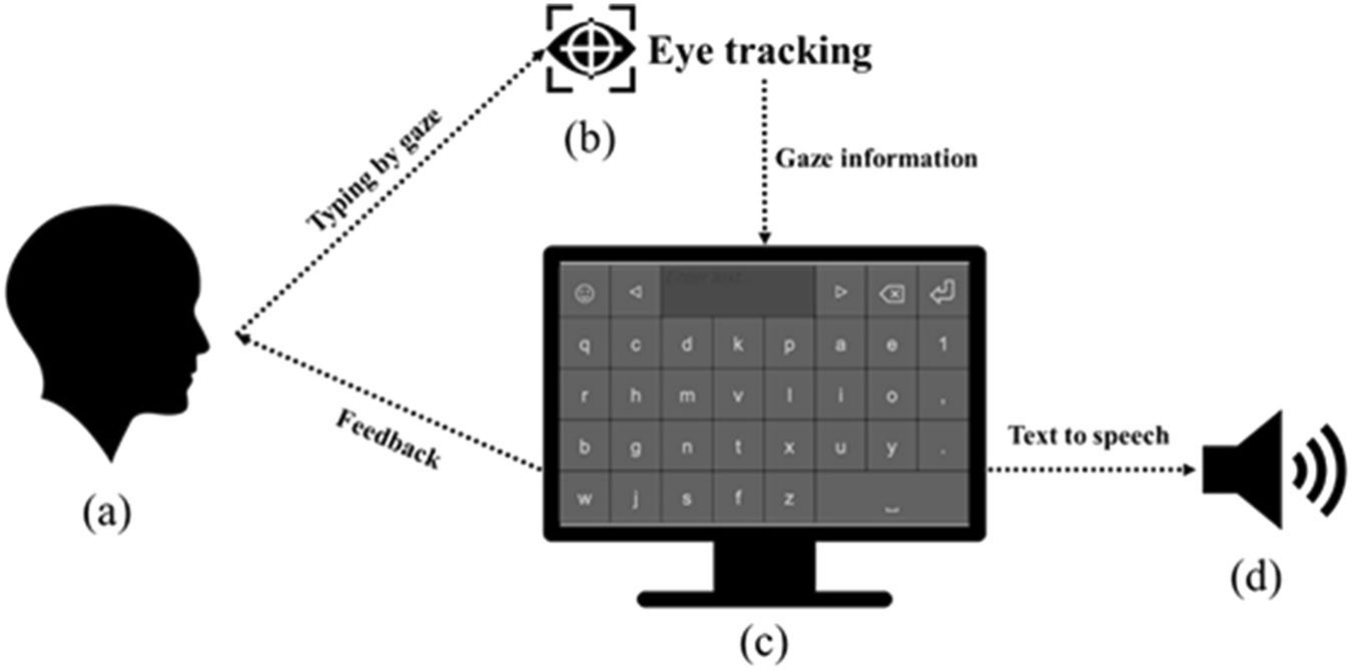
The eye-tracking based spelling communication system.

The spelling communication system employed in this study is an AAC system designed to aid Vietnamese individuals with impaired speech motor function, particularly those with ALS, in communicating with others by utilizing their eye gaze to type text messages through an on-screen keyboard^11^. The system, depicted in Fig. 1, is composed of an eye tracking device (b), a monitor for displaying an on-screen keyboard interface (c), a computer processor that is responsive to the eye tracking device, and a speaker (d). To use the system, the user (a) sits on a chair or lies on a specialized recliner and gazes at the on-screen keyboard displayed on the monitor, which is positioned approximately 80 cm in front of the user’s eyes. The eye tracking device (b), typically comprising one or two cameras located near the computer (usually below the monitor), tracks the user’s eye movements and provides the computer processor with the user’s gaze position. The system then analyzes this position information and determines the specific key on the on-screen keyboard (c) that the user is staring at and wishes to select. The system’s on-screen keyboard has a key layout and a quick typing suggestion mechanism specifically designed for the Vietnamese language. Additionally, the system can generate speech that corresponds to the text entered by the user and broadcast the sound to the speaker (d).

### Prerequisites for performing the study

Each participant provided voluntary written consent for their involvement in the data recording process in this study. For healthy individuals, data was recorded once in a spacious and enclosed laboratory. In the case of ALS patients, visits were scheduled every one to four weeks with the agreement of their caregivers, taking into account the patients’ health and well-being as well as resource optimization. However, due to the medical condition of one out of six patients and available resources, only one visit was conducted for this patient. For the remaining five patients, each received a total of ten visits. During each visit, a team of three members transported all equipment and set up all systems at the patient’s residence or lodging, taking into account the same criteria for the patient’s health and wellness. Additionally, all participants, including both ALS patients and healthy individuals, were trained to use the eye-tracking based spelling communication system, enabling them to form sentences by selecting letters using their eyes and communicate freely.

### Experimental paradigm

Prior to the recording experiment, participants were fully informed of the entire procedure, including an explanation of the steps and methods of the experiment, and all participants had a full understanding of the whole process. Meanwhile, the experimenter was responsible for supervising the experimental process to ensure its reliability. The data recording was conducted in a spacious and enclosed area. Before EEG/ET data acquisition, participants were required to perform eye movements as directed for ET calibration, and the experimenter help them to wear acquisition EEG equipment. During EEG/ET data acquisition, participants performed tasks as requested and instructed.

During one data collection experiment, each participant was engaged in nine sessions, each corresponding to a specific scenario related to a common human action or demand. These scenarios are described in Table 1.

**Table 1:** Spelling and movement tasks in the experimental paradigm.

No.	Task	Sentence in Vietnamese
1	Lifting the left hand	Nâng tay trái
2	Lifting the right hand	Nâng tay phải
3	Lifting the left leg	Nâng chân trái
4	Lifting the right leg	Nâng chân phải
5	Opening the mouth	Há miệng
6	Nodding the head	Gật đầu
7	Shaking head	Lắc đầu
8	Desiring to drink water	Tôi muốn uống nước
9	Desiring to use the bathroom	Tôi muốn đi vệ sinh

For scenarios 1 to 7, participants had to perform three types of tasks. First, they had to imagine the movement or demand corresponding to the scenario for a period of 5 to 7 seconds with closed eyes (task i). Then, they physically performed three consecutive movements corresponding to the scenario with open eyes and short 1–2 second rest between executions (task ii). Finally, they used the eye-tracking based spelling communication system to type the corresponding Vietnamese sentence related to the movement or demand (task iii). In these seven scenarios, participants alternated between tasks i and ii three times before proceeding to task iii. Participants with ALS performed the physical movements to the best of their ability considering their functional limitations. If they were unable to perform the movements, they refrained from doing so.

For scenarios 8 and 9, participants only performed tasks i three times before completing task iii (they did not perform task ii). Before starting each task, participants were given a 5-second resting interval for all nine scenarios.

**Fig. 2:**
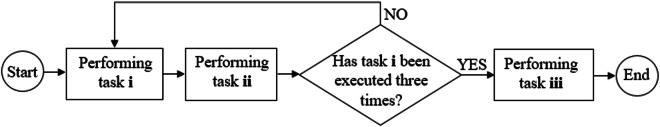
A recording session of a single participant.

Figure 2 illustrates a recording session of a participant for a scenario. Task i and ii were executed alternately three times, and finally, task iii was performed once. For scenarios 8 and 9, the participant did not perform task ii and executed task i three times in a row before performing task iii. During breaks between tasks, which lasted 5–7 seconds, the participant looked at the screen and rested their mind. The participant was asked not to perform any action unrelated to the experimental scenario being performed, such as leg shaking, arm shaking, stretching. The experimenter guided the participant to start or stop performing each task. EEG recordings were acquired during the execution of the task i and ii, while EEG and ET recordings were simultaneously obtained during the execution of the task iii. Each task execution would correspond to an event in the EEG signal.

### System for data acquisition

The EEG and ET data were acquired using a Recorder Software running on a Core i7 computer. This software, developed by our research team, enables simultaneous recording of both EEG and ET data. It has the capability to capture ET data while the user is utilizing the mentioned Spelling communication system. The EEG and ET data communicate through data stream LSL, which is a protocol that enables a streamlined and synchronized collection of time series measurements across multiple devices. Besides, the Recorder Software enables direct annotation of events which are mentioned in <*Experimental paradigm>* in the EEG signal.

**Fig. 3:**
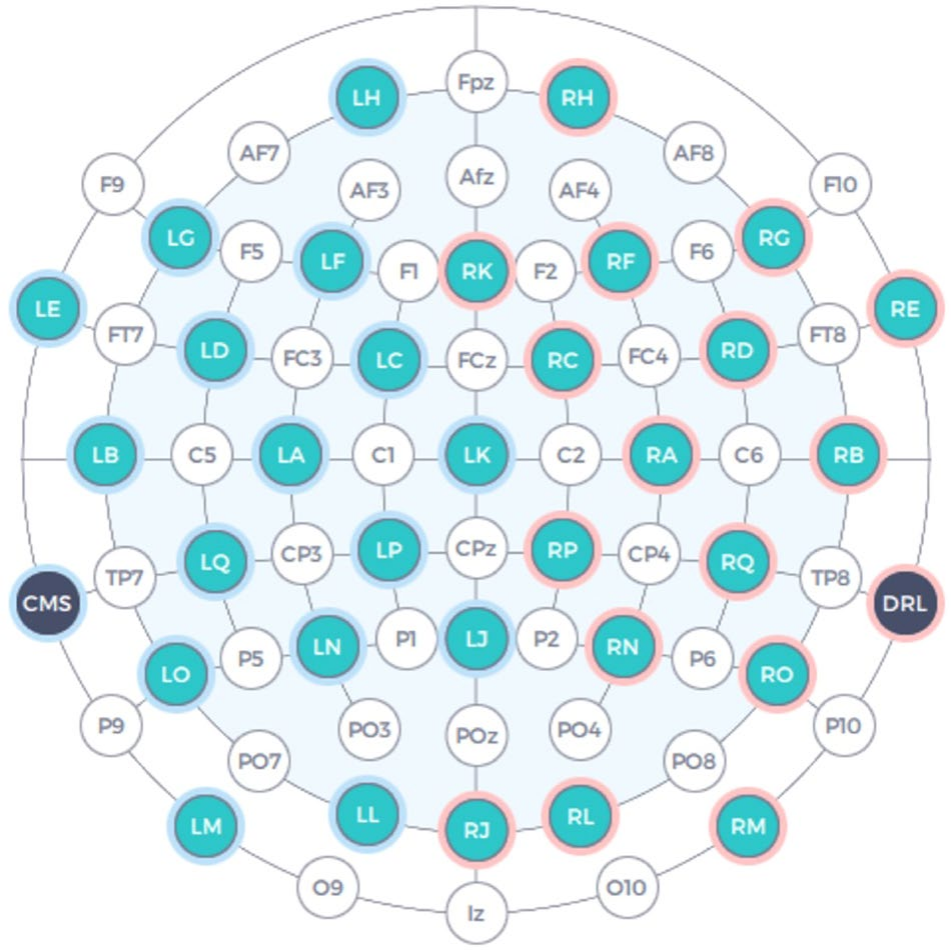
The position of EEG electrodes following the 10-10 standard.

To record EEG data, the Emotiv EPOC Flex^22^ device was used. It has 32 electrodes and they are installed by adapting the international standards 10–20^23^ with a sampling frequency of 128 Hz. We use saline sensors for the headset to prioritize participant comfort without compromising signal quality. Figure 3 illustrates the positions of the electrodes, with blue circles representing the positions for mounting the 32 electrodes in 10-10 system (extended from 10–20), and two black circles used for mounting the reference electrodes. ET data is captured by using Tobii Eye Tracker 5^24^ with a sampling frequency of 30 Hz.

## Data Records

### Raw data folders and usage notes

Our EEGET-ALS dataset^13^ is available at https://doi.org/XXXXX.

**Fig. 4:**
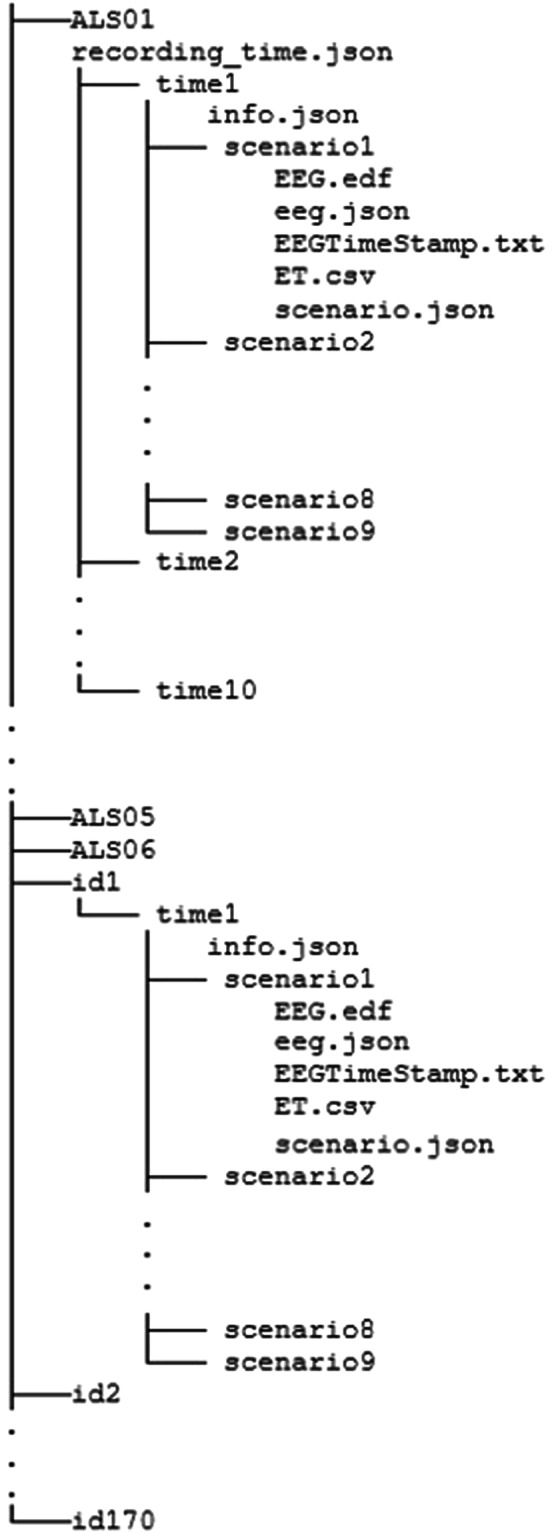
Structure of dataset directory.

In the EEGET-ALS dataset^13^, data was collected from each participant in 9 recording sessions, corresponding to 9 experimental scenarios, with each recording session lasts approximately 2 minutes. Each recording session includes events corresponding to 7 steps in one experimental scenario, and the participant was allowed to take short breaks (about 5 seconds) between steps to minimize fatigue. The data is organized into separate folders based on the participants’ identification codes. The structure of the dataset^13^ is depicted in Fig. 4. The root folder contains recorded data of 176 participants, with all personal identifying information removed. Data for each individual is stored in a separate subfolder which includes age and sex information, as well as data folders for different experimental scenarios, such as scenario 1, scenario 2, etc. The data folder for each scenario contains the following:

- Meta files (scenario.json, info.json) provide additional information of sample. scenario.json describes information about the recorded scenario while info.json presents information of participant i.e. sex, age…
- EEG data in EDF format (*EEG.edf*).
- A meta file (*eeg.json*) that provides information about the recorded EEG data, such as the number of channels and sampling rate.
- A meta file (*EEGTimeStamp.txt*) that stores the timestamps of the saved data in EEG.edf.
- ET data in CSV format (*ET.csv*).

The main raw data for each scenario is contained in *EEG.edf* and *ET.csv* files.

### EEG.edf

**Fig. 5:**
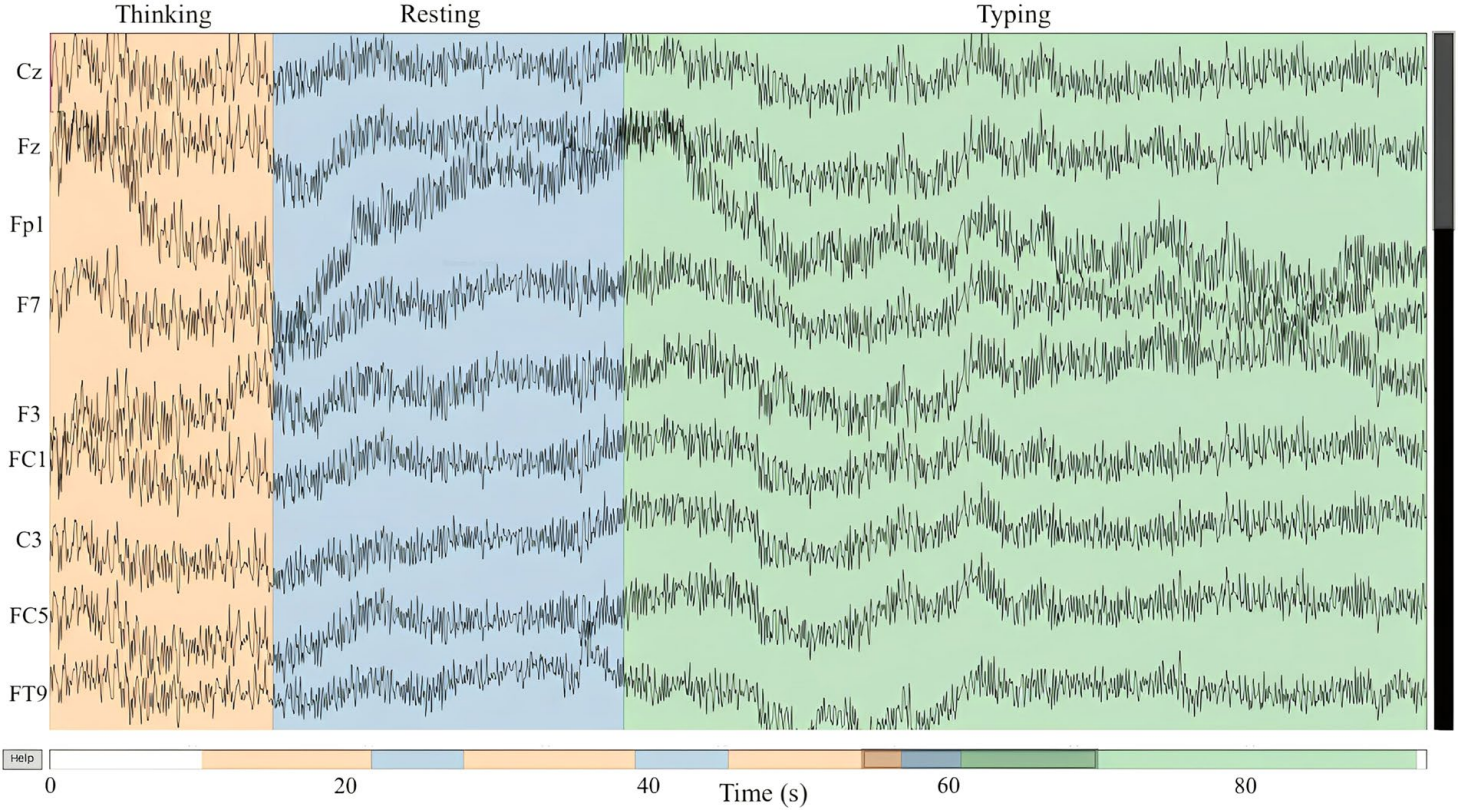
Preview data in an EEG.edf file by using MNE tool.

EEG file contains signal of 32 channels. The EEG data are stored in the European Data Format (EDF). Each EEG data file corresponds to a specific experimental scenario and contains multiple events and can be extracted using the MNE package for python^25^. These events correspond to the participant’s execution of tasks, as described in the <*Experimental paradigm>* section. Figure 5 provides an example of the different sessions in a data sample. In the Figure, the bottom bar represents the timeline of the recorded signal, with the current visualized signal highlighted in gray.

### ET.csv

**Fig. 6:**
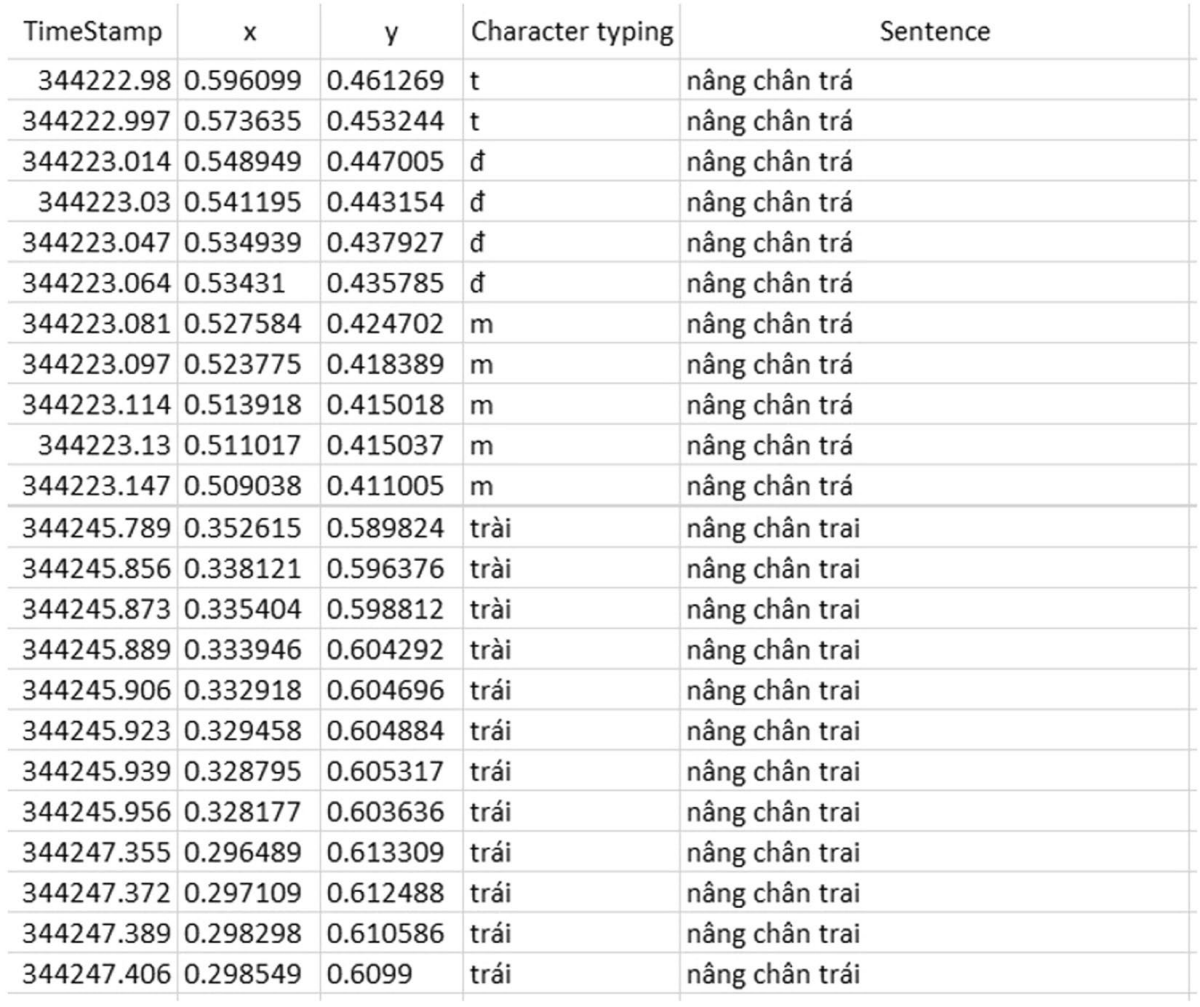
Sample of data in ET.csv.

Figure 6 shows sample of data in ET.csv. Each ET data frame is stored in a separate row which contains five fields of data, namely:

- *TimeStamp*: the time point of the recorded data frame; TimeStamp is in Unix time format.
- *x*: the x-coordinate that the user was looking at on the monitor of the eye-tracking based spelling communication system; the size of the monitor is 17 inches.
- y: the y-coordinate that the user was looking at on the monitor of the eye-tracking based spelling communication system.
- *character typing*: the letter or group of letters corresponding to the user’s eye gaze on the monitor of the eye-tracking based spelling communication system. The bounding coordinates for the on-screen key containing the letter or group of letters can be determined from the coordinates that the user was looking at on the monitor, using the supplied Python code in the “bb_determine.pdf” file.
- *sentence*: the characters that the user had typed.

For further information and details about the eye-tracking based spelling communication system, the layout of the on-screen keyboard, and how users interact with it, readers can refer to our earlier publication^11^.

For healthy participants, each session corresponding to a scenario was performed continuously in about from 2 to 3 minutes. The EEG and ET data of each session was recorded without interruption (meaning the EEG and ET data included the data during periods of rest between steps). All sessions were usually recorded consecutively over a period of approximately 40 minutes. Between sessions there was a period of approximately 30 seconds for participants to fully relax.

**Fig. 7:**
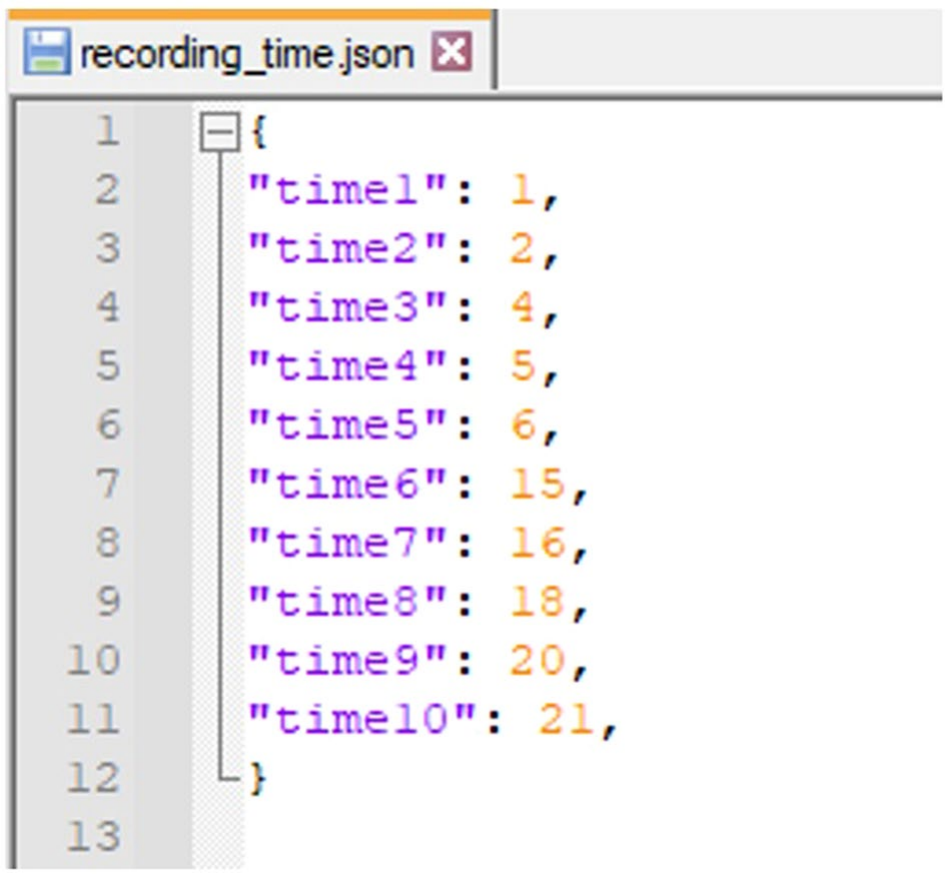
Example of description in file recording_time.json.

For 5 of 6 participants with ALS, we conducted 10 times of data acquisition (9 sessions each time same as for healthy subjects) over a period of 3 to 5 months. The recording times usually were 1 week apart, but due to some objective reasons such as the health status of the participants or the epidemic situation, some of the recording times were 2 to 4 weeks apart. Information for the recording time for each data acquisition time from 1 to 10 was described in a file ‘recording_time.json’; (Fig. 7 show an example of description in file ‘recording_time.json’). For this group of participants data, we named the data folder according to the syntax “ALS_[participant code]”. For the remaining participant with ALS, we were only able to perform 1 data collection time.

To facilitate ease of use, we have annotated certain events within the recorded data. Each annotation corresponds to one of the tasks (i, ii, iii, and “resting”) outlined in <*Experimental Paradigm>*, as detailed in Table 2.

**Table 2:** Annotations for certain events within the recorded data.

Task name	Task code	Annotation in EDF file
Imagination task	i	Thinking
Physical task	ii	Acting
Typing task	iii	Typing
Resting (between each task)	Resting	Resting

### Artifacts by physical tasks

Artifacts may be occurred when participants conduct the physical tasks. Users are advised to employ alignment and preprocessing methods when exploring our dataset to address these potential issues.

## Technical Validation

### EEG and ET recording setup

The participants were provided with specific instructions to reduce head movements during EEG and ET data collection. Furthermore, they were instructed to avoid any extraneous actions unrelated to the experimental scenario, such as leg or arm shaking or stretching, that could potentially introduce unwanted noise into the recordings. The recordings were conducted in a spacious, quiet and enclosed laboratory or room. To ensure consistency in recording conditions, the data were recorded at the same time intervals every day, from 8 AM to 11 AM and 1 PM to 5 PM.

### EEG Electrodes placement

The electrode placement adhered to the international 10-10 system, whereby 34 electrodes were positioned on the scalp, consisting of 32 electrodes for data acquisition and 2 for reference (Fig. 3). The electrode placement procedure was conducted by a qualified technician and validated by cross-checking against a scalp map. The reference electrode was located behind the participant’s ear.

### ET Calibration

Prior to data acquisition of EEG and ET, participants were required to move their eyes as instructed for ET calibration. Specifically, participants were directed to fixate on a series of calibration targets displayed on the monitor of the eye-tracking based spelling communication system. These calibration targets were presented at various locations on the screen to ensure precise gaze tracking across the entire display.

### Preprocessing steps

The raw EEG/ET data was provided without undergoing any preprocessing to ensure its integrity.

### Quality control measures

The Emotiv headset provided electrode impedance ratings from 0 (very bad) to 4 (very good), from which overall EEG quality was calculated as the average of the 3 three lowest rated channels, normalized to the maximum score of 4. The EEG electrode impedance was checked before and after each recording session to ensure electrode contact quality. If the overall quality computed based on three worst channels falls below 80, the recorded data is discarded. The raw EEG data was visually inspected to identify any clearly visible artifacts or noise originating from external environmental factors during recording. In case such noise or artifact was identified, the corresponding recording was eliminated from further analysis. The ET data was also visually inspected to ensure that the gaze data was accurate and free from artifacts. Please note that despite thorough checks, the distributed EEG/ET datasets may still contain some noise or artifacts, as only clearly visible issues could be removed. Users should take this into consideration when analyzing the data.

### Potential applications

The EEGET-ALS dataset^13^ collected in this study has the potential to be utilized for various research applications, such as determining the degree of participant attention to improve selection speed in gaze-controlled systems and for person identification (identifying the identity of an individual from a group of people).

To address the attention degree determination problem, we used the ET data to label the EEG data, which was processed to extract features for a determining classification. Attention data was defined as data collected when participants looked at a key to select it. Based on this definition, the EEG data was separated into two groups of samples: attention data (positive labeled) and inattention data (negative labeled) using ET data. We then extracted Power Spectral Density (PSD) and Common Spatial Patterns (CSP^26^) features from the EEG data and passed them to a simple classification such as Support Vector Machine (SVM) or Artificial Neural Networks (ANNs) to determine when the users were paying attention. This method with initial data had an accuracy of approximately 80% in the cross-check case.

In addition, person identification using EEG signals could improve user experience through personalization applications. To verify our data, we conducted several experiments on our recorded data for this problem. The EEG data was segmented and split into two sets as training and testing data. The learned model was then used to determine the identity of the testing signal. We employed four mechanisms for this investigation, including Support Vector Machine (SVM) learning and classification of the Power Spectral Density of the signal, SVM learning and classification of the features with inter-hemispheric amplitude ratio (IHAR^27^), Convolutional Neural Network (CNN) learning and classification of the raw signal, and Long short-term memory – Convolutional Neural Network (CNN-LSTM) learning and classification of the raw signal. The results provided in Table 3 demonstrate that the mentioned methods worked well on our EEGET-ALS dataset^13^.

**Table 3:** Person identification accuracy for four methods using our EEGET-ALS dataset (%).

Methods		All frequency band	4–8 Hz	8–13 Hz	13–30 Hz	30–50 Hz
Classification	Features					
SVM	PSD	88.20	77.74	82.64	82.14	70.18
SVM	IHAR	89.40	78.59	87.00	91.16	79.51
CNN	RAW	91.60	68.14	96.27	95.99	83.15
CNN-LSTM	RAW	94.51	80.38	98.58	98.33	94.04

**Fig. 8:**
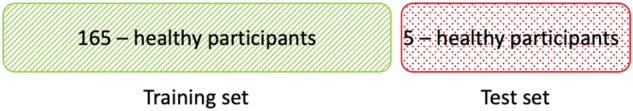
Train/test separation for experiment of only healthy participants.

**Fig. 9:**
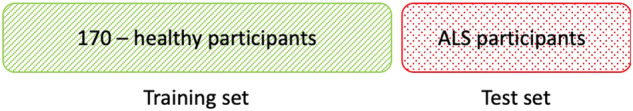
Train/test separation for experiment of healthy and ALS participants for MI classification.

Moreover, a common and essential task in the field of EEG is the classification of motor imagery (MI). MI classification involves the interpretation of EEG signals to identify and distinguish patterns related to imagined movements or actions. This task has applications in brain-computer interfaces (BCIs) and assistive technologies, where individuals can control devices or computers simply by thinking about specific motor actions. Accurate classification of motor imagery holds great promise in enhancing the quality of life for individuals with physical disabilities and advancing our understanding of brain function and control. To generate evaluation of MI classification on our dataset, 2 type of experiments settings which regarding to 2 parts in our datasets are conducted. This paper selected two classical algorithms (CSP^26^ and Euclidean Alignment - EA^28^) to enhance extracted informative features, and three machine learning or deep learning methods (SVM, EEG-ITNet^29^, and EEGNet^30^) for classification. Models are trained to predict 2 different sets of labels: 3 labels (LR0: lift left hand, lift right hand, resting) and 4 labels (LRF0: lift left hand, lift right hand, lift leg, resting). Performance of models are evaluated by 2 metrics: Balanced Accuracy (BAC) and Cohen’s Kappa (Kappa). The train/test separation for experiment of only healthy participants in our EEGET-ALS dataset^13^ is depicted in Fig. 8 and the corresponding MI classification results are presented in Table 4. The train/test separation for experiment of healthy and ALS participants in our EEGET-ALS dataset^13^ is illustrated in Fig. 9 and the corresponding MI classification results are presented in Table 5.

**Table 4:** MI classification results for 3 models on only healthy participants on our EEGET-ALS dataset.

	CSP-BP-SVM		[EA] EEGNet		[ЕА] EEG-ITNet	
	ВАС	Kарра	ВАС	Kарра	ВАС	Kарра
3 labels	0,6939	0,5882	0,6926	0,5705	0,7065	0,5949
4 labels	0,5518	0,5054	0,6028	0,5539	0,6043	0,5466

**Table 5:** MI classification results for EEG-ITNet model with EA technique on healthy and ALS participants on our EEGET-ALS dataset.

	ALS01		ALS02		ALS04		ALS*	
	ВАС	Kарра	ВАС	Kарра	ВАС	Kарра	ВАС	Kарра
3 labels	0,6466	0,435	0,5239	0,2695	0,5999	0,3605	0,59	0,3605
4 labels	0,4896	0,3293	0,3652	0,1526	0,3936	0,2077	0,4204	0,2422

(ALS* indicate the average number for all ALS participants)

For person identification, architecture of deep models are preserved as presented in papers^31^ with learning rate is 3e-4, batch size is 32 and Adam optimizer.

For MI classification e.g EEG-ITNET^30^ is trained with these hyperparameter: the size of batch is 16, the learning rate is 3e-4, maximum iteration times is 150 epoch, Adam optimizer is employed with objective function is cross entropy. Each EEG sample is cropped with a duration of 2 seconds.

### Supplementary information

HUMAN DATA SUBMISSION CHECKLIST

## Data Availability

• For the Motor Imagery task, code is available at: https://github.com/txdat/bci-motor-imagery/blob/master/notebooks/eeg_final.ipynb • For the person identification, code is available at: https://github.com/dangkh/VINIF_IdentifyPerson
